# C15-Structured Zr-Ti-Fe-Ni-V Alloys for High-Pressure Hydrogen Compression

**DOI:** 10.3390/ma18245482

**Published:** 2025-12-05

**Authors:** Jie Xu, Changsheng Qin, Hui Wang

**Affiliations:** 1 Guangdong Provincial Key Laboratory of Advanced Energy Storage Materials, School of Materials Science and Engineering, South China University of Technology, Guangzhou 510640, China; xu98525@163.com; 2 Guangxi Key Laboratory of Advanced Structural Materials and Carbon Neutralization, School of Materials and Environment, Guangxi Colleges and Universities Key Laboratory of Eco-Friendly Materials and Ecological Restoration, Guangxi Minzu University, Nanning 530105, China; qcs501@126.com

**Keywords:** hydrogen compression alloys, ZrFe_2_, alloying, quenching

## Abstract

Metal hydride hydrogen compressors (MHHC) offer unique advantages over conventional mechanical compressors in high-pressure hydrogen refueling. In this study, we developed C15-structured Zr-Ti-Fe-Ni-V single-phase alloys for high-pressure hydrogen compression. By designing the alloy compositions—high Ni and low V—and employing a quenching process, the resulting ZrFe_2_-based alloys exhibit reduced hydriding/dehydriding plateau hysteresis and slope, along with a narrow hydrogen solid solution zone. Notably, the Zr_0.8_Ti_0.2_Fe_1.2_Ni_0.7_V_0.1_ alloy elevates the hydrogen pressure from 128.3 atm to 334.5 atm within 283–353 K, delivering an effective hydrogen capacity of 1.02 wt.%. Similarly, the Zr_0.9_Ti_0.1_Fe_1.2_Ni_0.7_V_0.1_ alloy increases the hydrogen pressure from 60.4 atm to 221.8 atm across 283–363 K, with a capacity of 0.81 wt.%. This work provides a rational strategy for designing ZrFe_2_-based alloys for efficient hydrogen compression and storage applications.

## 1. Introduction

Hydrogen energy, as a clean and renewable energy carrier, plays a crucial role in achieving global carbon neutrality [1,2,3]. Because of its inherently low density under ambient temperature and pressure, hydrogen must be stored either at extremely high pressures or under low-temperature conditions to achieve a higher storage density [4,5]. Currently, commercial high-pressure hydrogen storage systems primarily employ diaphragm compressors for hydrogen refueling [6]. As an alternative to conventional mechanical compressors, metal hydride hydrogen compressors (MHHCs) exploit the unique property of hydrogen storage alloys, in which the hydrogen desorption plateau pressure increases with temperature to realize hydrogen pressurization. This method offers several notable advantages, including enhanced operational safety, lower maintenance costs and intrinsic hydrogen purification capability [7,8,9].

The core performance indicators of MHHCs are the hydrogen compression ratio and the effective hydrogen compression capacity, both of which are closely governed by the thermodynamic properties of the hydrogen storage alloys [10,11,12]. First, the hydrogen compression alloy should exhibit a low slope in the hydrogen absorption/desorption plateau, which facilitates a higher hydrogen compression ratio *R*_p_ = *P*_H_/*P*_L_, where *P*_L_ and *P*_H_ represent the right and left inflection points of the hydrogen absorption/desorption plateau on the pressure-composition isotherms (PCI), respectively. Second, the hydrogen compression alloy should possess a low hysteresis coefficient to reduce the energy consumption during cyclic hydrogen absorption and desorption. Furthermore, a high reversible hydrogen storage capacity further enhances the compression efficiency and hydrogen supply capacity. To ensure operational safety and energy efficiency, MHHC systems are typically designed to operate within the temperature range of 283–368 K, using water as the heat-transfer medium to regulate temperature. Depending on the design configuration, the required hydrogen compression ratios are approximately 80–250 atm for single-stage, 200–450 atm for double-stage, and 400–850 atm for three-stage compressors [13,14,15].

Currently, the most widely used hydrogen compression alloys in MHHC are LaNi_5_-based and TiCr_2_-based alloys [16,17,18,19,20]. LaNi_5_-based alloys exhibit several advantages, including fast hydrogen sorption kinetics, good cyclic stability, and strong resistance to impurity poisoning, making them particularly suitable for primary-stage hydrogen compression [16,17]. In contrast, the TiCr_2_-based alloys are mainly employed in intermediate and high-pressure hydrogen compression, typically operating within the range of 400–850 atm [13,18,19]. Compared with a TiCr_2_-based alloy that possesses a C14-type hexagonal Laves phase structure, a ZrFe_2_-based alloy with a C15 cubic structure demonstrates superior intrinsic properties, including ultra-high plateau pressures, flat plateau slopes and low *α*-phase hydrogen solubility [20,21]. These features are highly beneficial for achieving higher hydrogen compression ratios and greater effective hydrogen capacities. However, despite these advantages, the practical application of ZrFe_2_-based alloys in hydrogen compressors has been restricted due to their excessively high plateau pressures and pronounced hysteresis effects [22].

As is well known, hydrogen storage properties are fundamentally rooted in the interaction between their crystal structures and hydrogen atoms, manifesting as PCI curves and kinetics performance characteristics. Through the precise regulation of structural features and chemical composition, alloying enables synergistic improvements in the thermodynamics, kinetics, and cyclic stability of hydrogen storage, establishing it as the central strategy for optimizing the overall performance of hydrogen storage alloys. Therefore, to expand the applicability of ZrFe_2_-based alloys in hydrogen compressors, researchers have focused on reducing the hysteresis coefficient and plateau slope while fine-tuning the plateau pressure, primarily through elemental substitution for Fe [23,24,25,26,27,28]. For instance, the partial substitution of Fe with V effectively lowers the plateau pressure and mitigates hysteresis. However, excessive V addition leads to an increased plateau slope and a reduced hydrogen compression ratio [22,24]. Substituting Fe with Cr or Mn can decrease the plateau pressure of ZrFe_2_-based alloys while preserving a relatively flat plateau region [25,26]. Nevertheless, these alloying strategies often aggravate hysteresis, thereby decreasing the hydrogen compression ratio and increasing energy consumption. In addition, Ni—having a smaller atomic radius and a stronger affinity for hydrogen than Fe—can reduce the hysteresis coefficient of ZrFe_2_-based alloys while maintaining a low plateau slope [27]. However, when the Ni content is insufficient, the alloys tend to exhibit undesirable double plateaus on the PCI curves [28].

In this work, elemental substitutions of Ni, V, and Ti were introduced into ZrFe_2_, which originally has a high Ni content and low Ti/V ratio, to develop an intermediate hydrogen-compressing alloy with a C15 structure suitable for MHHC applications. Furthermore, a quenching process was employed to obtain a homogeneous single-phase structure with superior hydrogen sorption plateau characteristics, including a narrow solid-solution zone and a flat plateau. As a result, two Zr-Ti-Fe-Ni-V alloys exhibiting excellent hydrogen compression properties have been successfully developed.

## 2. Materials and Methods

### 2.1. Alloy Preparation

The series of Zr-Ti-Fe-Ni-V alloys (#1-Zr_0.8_Ti_0.2_Fe_1.15_Ni_0.7_V_0.15_, #2-Zr_0.75_Ti_0.25_FeNi_0.8_V_0.2_, #3-Zr_0.8_Ti_0.2_Fe_0.85_Ni_0.9_V_0.25_, #4-Zr_0.7_Ti_0.3_Fe_1.05_Ni_0.7_V_0.25_, #5-Zr_0.8_Ti_0.2_Fe_1.2_Ni_0.7_V_0.1_, #6-Zr_0.8_Ti_0.2_Fe_1.1_Ni_0.8_V_0.1_, #7-Zr_0.9_Ti_0.1_Fe_1.2_Ni_0.7_V_0.1_ and #8-Zr_0.7_Ti_0.3_Fe_1.2_Ni_0.7_V_0.1_) alloys were prepared by vacuum arc-melting in a DHL-1 vacuum-melting furnace. All the raw materials (Zr, Ti, Fe Ni and V ingots, over 99.9% purity) were purchased from Jiangxi GuoYan New Materials Co., Ltd. (Xinyu, China). A total of 10 g of raw materials was placed in the crucible, which was vacuumed to 2 × 10^−3^ Pa under an Ar atmosphere and then filled with 0.7–0.8 atm of high-purity argon. Each sample was smelted seven times for 20–30 s per cycle to ensure uniform alloy composition. The melt loss was kept below 0.5%. A portion of the molten alloy was polished and encased in a quartz tube, which was annealed at 1173 K and 1423 K for 5 days and then quenched in cool water; the heat treatment process diagram is shown in Figure S1. The hardened ingots were crushed and ground into powder after removing the oxide layer, and the alloy powder was then sieved through a 200-mesh screen for structural analysis and performance testing.

### 2.2. Structural Characterization

The phase structure of the alloys was analyzed by PANalytical Empyrean diffractometer (Malvern Panalytical, Malvern, UK), Cu-Ka radiation (2*θ* range: 20–100). The Rietveld structure refinement was performed by Fullprof software [29]. The field emission scanning electron microscope (SEM, TESCAN GAIA3, TESCAN, Brno, Czech Republic) equipped with an X-ray energy-dispersive spectrometer (EDS) was used to characterize the microstructure and composition of the samples.

### 2.3. Hydrogen Storage Measurement

The pressure-composition isotherms (PCI) were measured on a Sieverts-type hydrogen storage performance tester (Advanced Materials Corporation, Pittsburgh, PA, USA). High-purity hydrogen (99.999%) was used as the test gas. This device’s criteria for judging the equilibrium of each point in the PCI curve require one of the following two points: (a) the pressure fluctuation in the sample holder is less than 0.00049 psi/s; (b) the hydrogen was injected into the sample holder and held for more than 30 min. All samples were activated before PCI measurements by heating to 373 K and vacuumed for 30 min, then cooled to 223 K under 220 atm hydrogen and held for 10 h. PCI curves for hydrogen absorption and desorption were measured in a temperature range of 243–288 K and a pressure range between 100 atm and 0.06 atm.

## 3. Results and Discussion

### 3.1. Microstructures of Alloys

**Figure 1:**
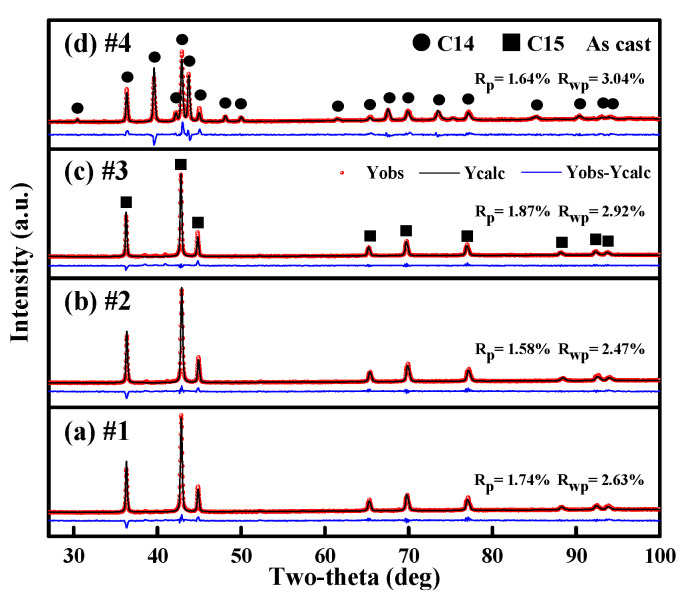
XRD patterns and Rietveld refinement patterns of the #1-Zr_0.8_Ti_0.2_Fe_1.15_Ni_0.7_V_0.15_ (**a**), #2-Zr_0.75_Ti_0.25_FeNi_0.8_V_0.2_ (**b**), #3-Zr_0.8_Ti_0.2_Fe_0.85_Ni_0.9_V_0.25_ (**c**), and #4-Zr_0.7_Ti_0.3_Fe_1.05_Ni_0.7_V_0.25_ (**d**) alloys.

Figure 1 shows the Rietveld refinement results of the XRD patterns for four as-cast alloys (#1-Zr_0.8_Ti_0.2_Fe_1.15_Ni_0.7_V_0.15_, #2-Zr_0.75_Ti_0.25_FeNi_0.8_V_0.2_, #3-Zr_0.8_Ti_0.2_Fe_0.85_Ni_0.9_V_0.25_ and #4-Zr_0.7_Ti_0.3_Fe_1.05_Ni_0.7_V_0.25_ alloys); the corresponding structural parameters are summarized in Table 1. It can be observed that all three alloys (#1–#3) crystallized in the C15 Laves phase with the space group *Fd-*3*m* (No. 227), whereas the #4 alloy adopts a hexagonal C14-type Laves phase with space group *P*6_3_/*mmc* (No. 194). The XRD patterns show that all four alloys are single-phase in nature.

As the Ti content increases from 0.2 to 0.25 and the Ni content decreases from 0.9 to 0.7, the alloys consistently exhibit a stable C15 single-phase structure. It is well known that the formation of Laves phases of AB_2_-type alloys is strongly influenced by the valence electron concentration (*e*/*a*) and the atomic radius ratio (*R*_A_/*R*_B_) [30]. Generally, a higher *e*/*a* value combined with a lower *R*_A_/*R*_B_ ratio tends to promote the structural transition from the C15 phase to the C14 phase. As shown in Table 1, the *e*/*a* and *R*_A_/*R*_B_ values of three alloys are #1 (1.58, 1.240), #2 (1.67, 1.234), #3 (1.75, 1.239), and #4 (1.75, 1.225). These results suggest that the formation of a C15 structure in ZrFe_2_-based alloys requires two critical conditions for value range of the *e*/*a* and *R*_A_/*R*_B_ ratio. Table S1 summarizes the Laves phase structures, valence electron concentrations (*e*/*a*), and atomic radius ratios (*R*_A_/*R*_B_) of large number of alloys across C15-type ZrFe_2_-based systems. Figure S2 illustrates the correlation between the C14 and C15 Laves phase structures of ZrFe_2_-based alloys and the two key parameters that *e*/*a* on the *x*-axis and *R*_A_/*R*_B_ on the *y*-axis. The observed structural trends are summarized as follows: (1) when *R*_A_/*R*_B_ < 1.22 or *e*/*a* > 2.43, the ZrFe2-based alloy stabilizes in the C14 phase; (2) when *R*_A_/*R*_B_ > 1.22 and *e*/*a* < 1.75, the ZrFe_2_-based alloy adopts the C15 phase; (3) when *R*_A_/*R*_B_ > 1.22 and 1.75 < *e*/*a* < 2.43, the ZrFe_2_-based alloy exhibits the coexistence of C14 and C15 phases. Therefore, the formation conditions of the C15 structure in Zr-Ti-Fe-Ni-V alloys are *R*_A_/*R*_B_ > 1.234 and *e*/*a* value < 1.75. This criterion is consistent with previous findings for other C15-structured Zr-Y-Fe and Zr-Fe-Al alloys [22,31].

**Table 1:** Structure parameters of #1–#4 alloys.

Alloys	*e*/*a*	*R*_A_/*R*_B_	Phase	*a* (Å)	*c* (Å)	*V* (Å^3^)
#1	1.58	1.240	C15	6.9994		342.91
#2	1.67	1.234	C15	6.9917		341.78
#3	1.75	1.239	C15	7.0053		343.78
#4	1.75	1.225	C14	4.9453	8.0605	170.72

Where *e*/*a* = *Z*_1_*C*_1_ + *Z*_2_*C*_2_ + … + *Z*_m_*C*_m_, *Z_i_* (*i* = 1 − *s*) is the number of valence electrons of element *i*, *C_i_* is the atomic fraction of element *i*, and *C*_1_ +… + *C*_m_ = 1. The valence electron number of metallic elements is the number of atomic groups, but the valence electron number of VIIIB group elements such as Fe and Ni is zero [32]. *R*_A_ = *R*_1_*X*_1_ + *R*_2_*X*_2_ + … + *R*_m_*X*_m_ (*R*_B_ = *R*_a_*Y*_a_ + *R*_b_*Y*_b_ + … + *R*_n_*Y*_n_), *R*_i_ (*i* = 1 − m) is the atomic radius of the A-side element; *R*_j_(*j* = 1 − n) is the atomic radius of the B-side element; and *X*_1_ + *X*_2_ +… + *X*_m_ = 1, *Y*_1_ + *Y*_2_ +… + *Y*_n_ = 1.

**Figure 2:**
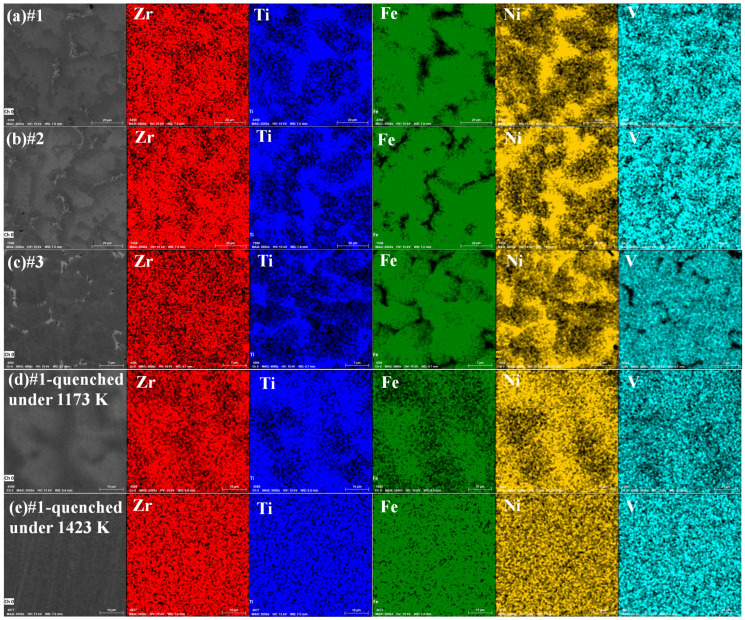
Backscattering SEM morphologies and elemental mappings of #1-Zr_0.8_Ti_0.2_Fe_1.15_Ni_0.7_V_0.15_ (**a**), 2-Zr_0.75_Ti_0.25_FeNi_0.8_V_0.2_ (**b**), #3-Zr_0.8_Ti_0.2_Fe_0.85_Ni_0.9_V_0.25_ (**c**), and the Zr_0.8_Ti_0.2_Fe_1.15_Ni_0.7_V_0.15_ alloys quenched at 1173 K (**d**) and 1423 K (**e**).

Figure 2a–c show the SEM images and EDS elemental mappings of the as-cast alloys. Although the XRD results show that all three alloys (#1–#3) crystallize in a single C15 phase, distinct phase contrast variations are visible in the backscattered SEM images. These differences in contrast are associated with the non-uniform distribution of Zr, Ti, Fe, and Ni elements, as confirmed by the elemental mappings. Specifically, Fe does not fully dissolve with Ti and Ni in the as-cast alloys, resulting in marked compositional inhomogeneity. This is primarily attributed to the wide solid solubility range of ZrFe_2_ phase, in which moderate compositional fluctuations do not induce the precipitation of a secondary phase. A similar phenomenon has also been observed in studies of ZrFe_2_-type Zr-Ti-Fe-Mn alloys [33].

To optimize the microstructure, the #1 alloy (Zr_0.8_Ti_0.2_Fe_1.15_Ni_0.7_V_0.15_) was selected for quenching treatments at different temperatures. After quenching at 1173 K, Figure 2d shows a slight phase-contract difference but improved elemental uniformity. When the quenching temperature is increased to 1423 K, the alloy exhibits a completely homogeneous elemental distribution and a well-defined single-phase microstructure. This is caused by the increasing atomic diffusion energies of Zr, Ti, Fe and Ni elements at elevated temperatures, which promotes atomic mobility. Additionally, high-temperature annealing reduces lattice defects and creates more pathways for atomic diffusion.

**Figure 3:**
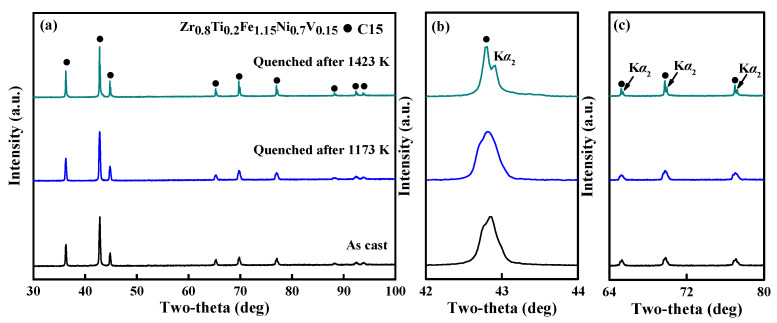
XRD patterns of Zr_0.8_Ti_0.2_Fe_1.15_Ni_0.7_V_0.15_ alloys at different quenching treatments with 2 theta range of 30–100° (**a**), 42–44° (**b**), and 64–80° (**c**).

The XRD patterns of the quenched alloys (Figure 3a) confirm that the C15 structure is retained in all samples. A close examination of the enlarged profiles (Figure 3b,c) shows that the broadened peaks in both the as-cast alloy and the 1173 K-quenched alloy correspond to overlapping C15 phases with a slightly different composition, as illustrated in Figure 2. In contrast, the 1423 K-quenched alloy displays sharp and well-resolved diffraction peaks, where each distinct *K*_α1_ peak is accompanied by a clearly separated *K*_α2_ peak with an intensity ratio of approximately 2:1.

This result provides further evidence of enhanced crystallinity and the successful formation of a uniform single-phase structure at higher quenching temperatures. Overall, these findings demonstrate that the quenching process effectively refines the microstructure of multicomponent Zr-Fe-Ni-Ti-V alloys, promoting compositional homogeneity and structural stability.

### 3.2. Hydrogen Storage Properties

**Figure 4:**
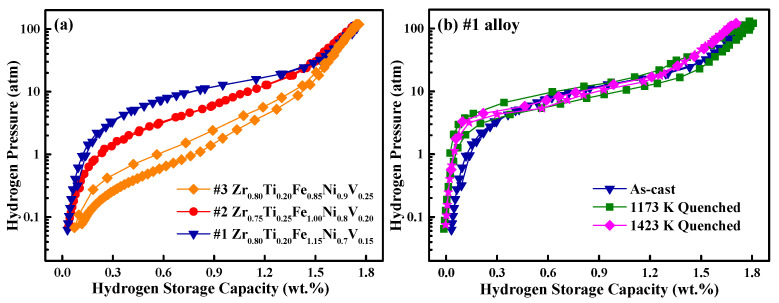
PCI curves at 243 K for #1–#3-Zr-Ti-Fe-Ni-V alloys (**a**) and a comparison of #1 alloy when as-cast, 1173 K-quenched, and 1423 K-quenched (**b**).

The PCI curves tested at 243 K of the #1–#3 as-cast alloys, and the quenched #1 alloy are shown in Figure 4, with corresponding hydrogen absorption/desorption performance data summarized in Table 2. The #1-Zr_0.8_Ti_0.2_Fe_1.15_Ni_0.7_V_0.15_ alloy exhibits higher hydrogen storage capacity and plateau pressure than both the #2-Zr_0.75_Ti_0.25_Fe_1_Ni_0.8_V_0.2_ and #3-Zr_0.8_Ti_0.2_Fe_0.85_Ni_0.9_V_0.25_ alloys. The content of elements in alloys has a direct impact on hydrogen storage performance. The elevated V and Ni contents are the main reasons contributing to the decreased hydrogen storage capacity and plateau pressure [28,34]. In addition, both #1 and #2 alloys exhibit significantly low *H_f_* values, and their hydrogen absorption/desorption PCI curves are almost overlapped. However, the *H_f_* value of #3 alloy slightly increases up to 0.5 with increasing Ni content, potentially due to the excessive Ni content and the strong affinity between Ni and H [35]. Notably, the *S_f_* value of all the three as-cast alloys exceeds 1.5, which may be attributed to the heterogeneous microstructure and inhomogeneous compositions [36]. Therefore, the Ni content should be maintained below 0.9, while the V content should not exceed 0.15.

Figure 4b compares the PCI curves of the #1 alloy (Zr_0.8_Ti_0.2_Fe_1.15_Ni_0.7_V_0.15_) before and after quenching. The hydrogen storage capacity shows a slight increase after quenching at 1173 K but a decrease when quenched at 1423 K. This is because the elemental distribution in the alloy evolved from three distinct regions to two after quenching at 1173 K, indicating enhanced compositional homogenization contributes to an improved hydrogen storage capacity. As temperature further increased to 1423 K, the alloy achieved full elemental uniformity, accompanied by the elimination of structural defects. However, this reduction in defect density and phase boundary interfaces diminished the number of available sites for hydrogen atom accommodation, leading to a decrease in hydrogen storage capacity. Furthermore, both quenched alloys demonstrated reduced desorption plateau pressures compared to the as-cast alloy, indicating that the more homogeneous composition achieved after quenching optimizes the diffusion pathways of hydrogen atoms, reducing the diffusion energy barrier and thus decreasing the hydrogen desorption plateau pressure. The 1423 K-quenched alloy displays a lower hydrogen absorption pressure but a higher desorption pressure compared with the 1173 K-quenched sample, resulting in a lower hysteresis factor (*H_f_* = 0.12). The quenching process significantly enhances compositional homogeneity and plateau features, as evidenced by the narrowing of the hydrogen solid solution zone (*C_s_*) from 0.29 wt.% in the as-cast state to 0.09 wt.% after quenching at 1423 K. This is primarily attributed to the wide solid solubility range of the ZrFe_2_ phase, which accommodates moderate compositional fluctuations without inducing secondary phase precipitation. High-temperature annealing further contributes by reducing lattice defects and facilitating atomic diffusion through the creation of additional diffusion pathways. As shown in Figure 2, when the #1 alloy was quenched at 1173 K, the element distribution changed from three distinct regions to two, indicating a gradual homogenization. When the temperature was raised to 1423 K, the alloy achieved complete elemental homogeneity. The resulting compositional optimization improves hydrogen diffusion kinetics by providing more coherent diffusion channels, thereby yielding a flatter desorption plateau and reduced hysteresis. This improvement indicates that the quenching process effectively increases the hydrogen compression efficiency [39]. Compared with other C15 structure alloys, all the Zr-Ti-Fe-Ni-V alloys studied in this study for hydrogen compression have a flatter hydrogen absorption/desorption plateau, lower hysteresis, and a similar slope factor [22,31,33].

Therefore, these results demonstrate that a relatively high Ni content and low V content in ZrFe_2_-based alloys are beneficial for improving the slope factor and hysteresis factor of the hydrogen absorption/desorption plateaus. The primary role of V lies in enhancing structural stability through electron hybridization, thereby improving hydrogen storage capacity and diffusion kinetics. In contrast, Ni functions by forming intermetallic compounds and regulating phase structure, which enables a balanced trade-off between hydrogen storage capacity and cycling stability, thus facilitating performance optimization in multi-component alloys [40]. Accordingly, a quenching temperature of 1423 K was selected as the optimal condition for fabricating single-phase C15-structured alloys with enhanced hydrogen storage and compression properties.

### 3.3. Optimization of Alloy’s Composition and Hydrogen Storage Performance

To further improve the plateau characteristics, four modified alloys with reduced V content were designed based on the #1 alloy’s composition (Zr_0.8_Ti_0.2_Fe_1.15_Ni_0.7_V_0.15_). These alloys, #5 (Zr_0.8_Ti_0.2_Fe_1.2_Ni_0.7_V_0.1_), #6 (Zr_0.8_Ti_0.2_Fe_1.1_Ni_0.8_V_0.1_), #7 (Zr_0.9_Ti_0.1_Fe_1.2_Ni_0.7_V_0.1_), and #8 (Zr_0.7_Ti_0.3_Fe_1.2_Ni_0.7_V_0.1_), were developed by optimizing the Ni and Ti contents. All samples were prepared via quenching at 1423 K to ensure compositional homogeneity.

The XRD patterns and SEM images (Figures S3 and S4) confirm that all four alloys possess a single C15 phase with a uniform elemental distribution. As summarized in Table 3, the cell volume shows a slight decrease from 341.92 Å^3^ to 341.22 Å^3^ with increasing Ni content (from 0.7 to 0.8). In contrast, a more significant reduction in cell volume, from 345.55 Å^3^ to 338.23 Å^3^, is observed as the Ti content rises from 0.1 to 0.3. These variations in cell volume directly correlate with the elevation of plateau pressure in the alloys, suggesting that atomic substitution plays a key role in tuning the thermodynamic properties of the C15 structure.

**Figure 5:**
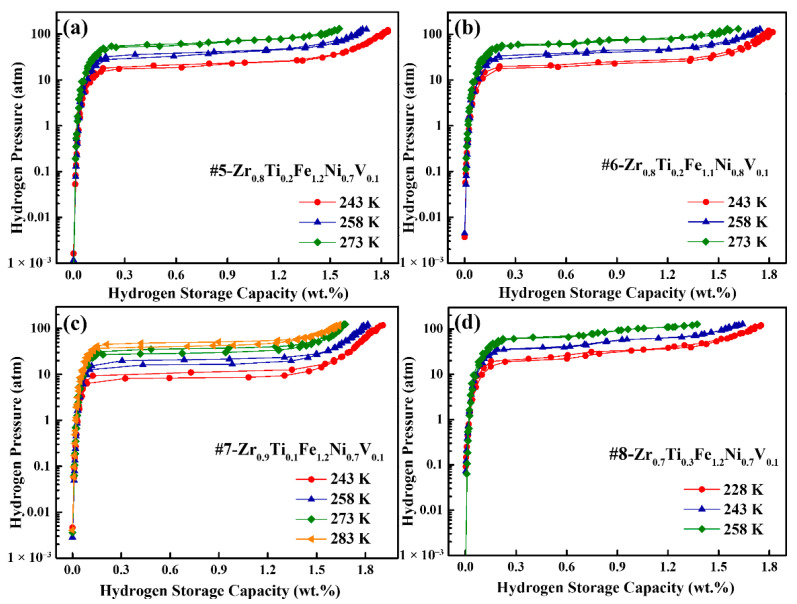
PCI curves of (**a**) #5-Zr_0.8_Ti_0.2_Fe_1.2_Ni_0.7_V_0.1_, (**b**) #6-Zr_0.8_Ti_0.2_Fe_1.1_Ni_0.8_V_0.1_, (**c**) #7-Zr_0.9_Ti_0.1_Fe_1.2_Ni_0.7_V_0.1_ and (**d**) #8-Zr_0.7_Ti_0.3_Fe_1.2_Ni_0.7_V_0.1_ alloys at different temperatures.

The PCI curves of the #5–#8 alloys measured at different temperatures are shown in Figure 5, and the corresponding hydrogen storage properties are summarized in Table 3. All alloys exhibit distinct and well-defined hydriding/dehydriding plateaus, accompanied by complete hydrogen release upon dehydrogenation. It is also noted that these alloys exhibit narrow solid solution zones, which help to improve the effective hydrogen compression capacity. Among the tested compositions, the #7 alloy (Zr_0.9_Ti_0.1_Fe_1.2_Ni_0.7_V_0.1_) exhibits the highest capacity (1.90 wt.%) at 243 K, while the #8 alloy (Zr_0.7_Ti_0.3_Fe_1.2_Ni_0.7_V_0.1_) shows the lowest capacity (1.75 wt.%) at the same temperature, suggesting a correlation between hydrogen storage capacity and absorption plateau pressures.

As expected, the plateau pressures increase with higher Ni and Ti contents. The hydrogen absorption and desorption pressures gradually increase from 23.20/20.64 atm (#5) to 26.71/22.03 atm (#6) as the Ni content rises from 0.7 to 0.8. In contrast, the plateau pressures for the alloys with varying Ti content exhibit a more pronounced increase. Specifically, the hydrogen absorption and desorption pressures rise sharply from 11.82/8.50 atm (#7, Ti = 0.1) to 33.25/30.07 atm (#8, Ti = 0.3) at 243 K. This substantial difference highlights the significantly greater impact of Ti content on plateau pressure compared to the influence of Ni content.

Compared with the PCI curve of #1 alloy (Zr_0.8_Ti_0.2_Fe_1.15_Ni_0.7_V_0.15_) at 243 K in Figure 4, the PCI curves of the #5–#8 alloys at same temperature show a significantly reduced plateau slope, while maintaining a consistently low hysteresis factor. Among them, the #7 alloy (Zr_0.9_Ti_0.1_Fe_1.2_Ni_0.7_V_0.1_) shows a notably low slope factor of 0.19 at 243 K, which is much lower than the value of 1.59 observed for the #1 alloy. The *S_f_* value decreases slightly with increasing Ni content but increases with higher Ti content. In addition, the *H_f_* value increases from 0.10 for the Ti = 0.3 alloy to 0.33 for the Ti = 0.1 alloy. This trend is consistent with previous observations reported for Zr-Fe-Mn-V alloys [33].

**Figure 6:**
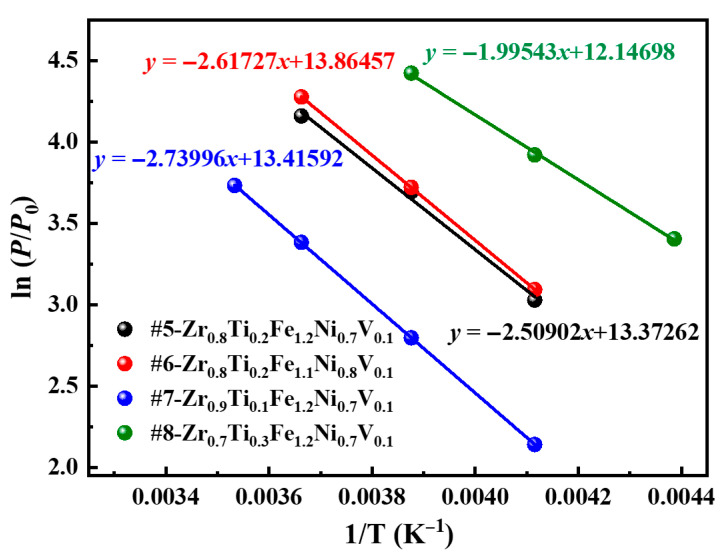
van’t Hoff plots of four alloys.

Figure 6 shows the van’t Hoff curves of four alloys, derived from the thermodynamic relation: ln (*P*/*P*_0_) = Δ*H*/*RT* − Δ*S*/R [41]. In this equation, *R* denotes the universal gas constant and *T* represents the absolute temperature in Kelvin. The data reveals a strong linear correlation between ln*P* and 1/*T*, which enables the determination of the enthalpy change (Δ*H*_d_) and entropy change (Δ*S*_d_) associated with hydrogen desorption. The calculated values are summarized in Table 3. The Δ*H*_d_ values exhibit a slight increase with increasing Ni content, rising from 20.86 kJ/mol for the #5 alloy (Ni = 0.7) to 21.76 kJ/mol for the #6 alloy (Ni = 0.8). Conversely, the Δ*H*_d_ decreases with increasing Ti content, dropping from 22.78 kJ/mol for the #6 alloy (Ti = 0.1) to 16.59 kJ/mol for the #8 alloy (Ti = 0.3). This trend reflects the strong hydrogen affinity of Ti, which reduces the stability of the hydride phase and consequently lowers the desorption enthalpy. Furthermore, these alloys demonstrate a lower hydrogen desorption enthalpy relative to Zr-Y-Fe [31] and Zr-Ti-Fe-Mn [33] alloys, which reduces the thermodynamic stability of the hydride phase and facilitates more efficient hydrogen release during the decompression process. Based on the fitted van’t Hoff plots, the extrapolated hydrogen desorption plateau pressures of the four alloys range from 67 atm to 70 atm at 298 K, and from 286.7 atm to 677.6 atm at 353 K. These results confirm the excellent potential of the developed alloys for high-pressure hydrogen compression applications.

Moreover, the XRD patterns of all the #5–#8 alloys after hydrogen absorption and desorption cycle are shown in Figure S5, and the corresponding cell parameters summarized in Table S2. All the alloys retain the C15 single-phase structure after hydrogen absorption and desorption cycle, as evidenced by the sharp and intense XRD diffraction peaks in Figure S4. The minimal change in cell volume before and after PCI measurements further supports its structural stability. Notably, the #8 alloy exhibits a mere 0.047% cell volume contraction after hydrogen desorption, confirming the robustness of the crystal structure during hydrogen absorption/desorption cycles.

### 3.4. Hydrogen Compression Performance

**Figure 7:**
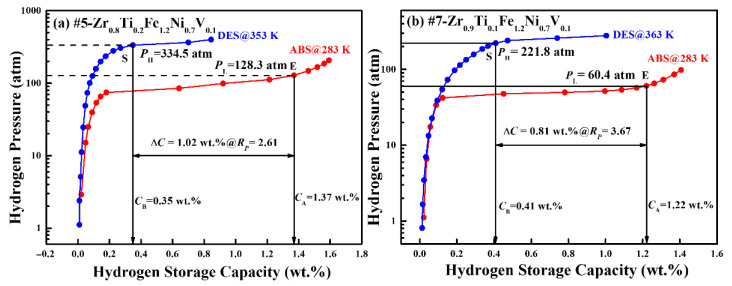
Measured PCI curves of 5#-Zr_0.8_Ti_0.2_Fe_1.2_Ni_0.7_V_0.1_ (**a**) and #7-Zr_0.9_Ti_0.1_Fe_1.2_Ni_0.7_V_0.1_ (**b**) alloys at working temperatures.

The #5 alloy (Zr_0.8_Ti_0.2_Fe_1.2_Ni_0.7_V_0.1_) and #7 alloy (Zr_0.9_Ti_0.1_Fe_1.2_Ni_0.7_V_0.1_) were selected for PCI measurement under optimized working conditions, with test temperatures ranging from 283 K to 363 K and a maximum hydrogen pressure of 400 atm. The PCI curves are shown in Figure S6. The hydrogen absorption PCI curve was first recorded at the lower temperature (*T*_L_), followed by the desorption PCI measured at the higher temperature (*T*_H_). The resulting PCI curves for both alloys are shown in Figure 7, and the corresponding hydrogen compression performance data are summarized in Table 4.

In these PCI curves, the endpoint (*E*) of the hydrogen absorption plateau at *T_L_* is adopted to determine the hydrogen absorption capacity (*C_A_*) and the input pressure (*P*_L_) for the MHHC process, while the point (*S*) on the hydrogen desorption plateau at *T_H_* corresponds to the remained capacity (*C_B_*) and output pressure (*P*_H_). The reversible hydrogen capacity (*C_c_* = *C_A_–C_B_*) and the compression ratio (*R_p_* = *P*_H_/*P*_L_) are thus obtained, reflecting the effective hydrogen storage capacity and the cyclic efficiency of hydrogen compression [42,43,44].

The #5 alloy (Zr_0.8_Ti_0.2_Fe_1.2_Ni_0.7_V_0.1_) exhibits a hydrogen absorption capacity of 1.37 wt.% at 283 K under hydrogen pressure of 128.3 atm. It provides a reversible 1.02 wt.% of hydrogen above a hydrogen pressure of 334.5 atm at 353 K, corresponding to a hydrogen compression ratio of 2.61. In comparison, the #7 alloy (Zr_0.9_Ti_0.1_Fe_1.2_Ni_0.7_V_0.1_) delivers a hydrogen absorption capacity of 1.22 wt.% at 283 K under 60.4 atm, and releases 0.81 wt.% of hydrogen at 363 K and 221.8 atm, achieving a higher hydrogen compression ratio of 3.67. Both Zr-based alloys exhibit a hydrogen compression performance comparable to or even superior to those of previously reported C14-structured Ti-based alloys, as summarized in Table 4. For instance, Peng et al. [42] developed a Ti_1.08_Cr_1.3_Mn_0.2_Fe_0.5_ alloy, which is capable of compressing hydrogen from 237.4 atm to 535.2 atm within the temperature range of 298–363 K, achieving an effective hydrogen compression capacity of 0.66 wt.% and a compression ratio of 2.25. Therefore, the present ZrFe_2_-based alloys achieve comparable or higher compression ratios (up to 3.67), together with larger reversible hydrogen capacities (>0.8 wt.%), highlighting their strong potential for practical applications in MHHC.

The superior hydrogen compression performance of these ZrFe_2_-based alloys can be primarily attributed to their C15 cubic Laves phase structure, which possesses higher crystallographic symmetry and a more isotropic lattice compared with C14 hexagonal structure. This structural characteristic effectively minimizes local stress concentration during hydrogen absorption and desorption, thereby suppressing lattice distortion and improving plateau characteristics. In addition, the quenching process plays a key role in promoting compositional homogeneity and suppressing secondary phase formation, which further reduces hysteresis and enhances plateau flatness. Further, the combined effect of high Ni and low V contents contributes to the flattening of the plateau and the narrowing of the solid solution region, both of which are essential for achieving a high hydrogen compression ratio.

From an application standpoint, these findings suggest that the rational design of C15-type multicomponent alloys through compositional optimization and thermal treatment can yield high-performance hydrogen compression materials. The obtained hydrogen compression ratios (2.61–3.67) and reversible capacities (0.81–1.02 wt.%) meet or exceed the operational requirements for medium- and high-pressure stages in multi-stage MHHC. Moreover, the narrow solid solution zone and low hysteresis ensure minimal energy loss and long-term reliability.

## 4. Conclusions

This study investigates the effects of elemental substitution (Ti, Ni and V) and the quenching process on the microstructure and hydrogen compression performance of C15-structured ZrFe_2_-based alloys. The key findings are summarized as follows: 1. a high Ni content combined with low V content effectively reduce the hysteresis effect and improve the plateau slope; 2. the quenching process promotes the formation of a single-phase structure with uniform composition, improving plateau characteristics while narrowing the hydrogen solid solution zone; 3. the Zr_0.8_Ti_0.2_Fe_1.2_Ni_0.7_V_0.1_ alloy can increase the hydrogen pressure from 128.3 atm to 334.5 atm with an effective hydrogen compression capacity of 1.02 wt.% within the temperature range of 283–353 K. The Zr_0.9_Ti_0.1_Fe_1.2_Ni_0.7_V_0.1_ alloy can increase the hydrogen pressure from 60.4 atm to 221.8 atm within the temperature range of 283–363 K, providing an effective hydrogen compression capacity of 0.81 wt.%. These results emphasize the significant potential of ZrFe_2_-based alloys for hydrogen compression applications.

## Figures and Tables

**Table 2:** Hydrogen storage thermodynamics properties of alloys at 243 K.

Alloys	*C*_m_ (wt.%)	*C*_s_ (wt.%)	*P*_a_ (atm)	*P*_d_ (atm)	*H_f_*	*S_f_* (*P*_d_)
#1 alloy	1.78	0.29	10.01	9.84	0.02	2.18
#2 alloy	1.74	0.25	6.06	5.99	0.01	2.33
#3 alloy	1.74	0.18	2.25	1.33	0.53	3.07
#1-1173 K-quenched	1.81	0.07	10.95	6.94	0.23	1.35
#1-1423 K-quenched	1.71	0.09	9.26	8.21	0.12	1.59

*C*_m_: Hydrogen absorption capacity. *C*_s_: The hydrogen storage capacity corresponding to the solid solution region. *P*_a_: the pressure corresponding to half of the hydrogen absorption capacity of the PCI curve. *P*_d_: the pressure corresponding to half of the hydrogen release capacity of the PCI curve. *H_f_*: defined as ln (*P*_a_/*P*_d_) [37], hysteresis coefficient of hydrogen release curve. *S_f_*: defined as ln (*P*_75%_/*P*_15%_) [38], plateau slope of hydrogen release curve, *P*_75%_ and *P*_15%_ refers to plateau pressure corresponding to 75% and 15% hydrogen desorption capacity.

**Table 3:** Cell volume and hydrogen storage properties of alloys quenched at 1423 K.

Alloys	*V* (Å^3^)	*T* (K)	*C*_m_ (wt.%)	*P*_a_ (atm)	*P*_d_ (atm)	*H_f_*	*S_f_*	Δ*H*_d_ (kJ/mol)	Δ*S*_d_ (J/mol K)
#5	341.92	243	1.84	23.20	20.64	0.12	0.41	20.86	111.18
		258	1.71	42.66	40.18	0.06	0.48	±1.428	±5.553
		273	1.55	69.18	64.01	0.08	0.50		
#6	341.22	243	1.82	26.71	22.03	0.19	0.35	21.76	115.27
		258	1.74	45.96	41.29	0.11	0.41	±0.043	±0.167
		273	1.62	76.19	71.95	0.06	0.47		
#7	345.55	243	1.90	11.82	8.50	0.33	0.19	22.78	111.54
		258	1.81	21.22	16.38	0.26	0.19	±0.046	±0.176
		273	1.67	36.37	29.44	0.21	0.06		
#8	338.23	228	1.75	33.25	30.07	0.10	0.66	16.59	100.99
		243	1.64	51.04	50.41	0.01	0.49	±0.452	±1.868
		258	1.37	85.35	83.27	0.02	0.63		

**Table 4:** Comparison of hydrogen compression properties for different alloys.

Alloys	Temperature (K)	*P*_L_ (atm)	*P*_H_ (atm)	*R*_p_	*C*_c_ (wt.%)	Ref.
#5-Zr_0.8_Ti_0.2_Fe_1.2_Ni_0.7_V_0.1_	283/353	128.3	334.5	2.61	1.02	This work
#7-Zr_0.9_Ti_0.1_Fe_1.2_Ni_0.7_V_0.1_	283/363	60.4	221.8	3.67	0.81	This work
Ti_1.08_Cr_1.3_Mn_0.2_Fe_0.5_	298/363	237.4	535.2	2.25	0.66	[42]
Ti_0.8_Zr_0.2_Cr_0.95_Fe_0.95_V_0.1_	298/423	385	745	1.94		[43]
Ti_0.86_Mo_0.14_Cr_1.9_	293/363	830.9	1759	2.12		[44]
ZrFe_1.8_Ni_0.2_	293/363	461	922	2.00		[44]

*C_c_* = *C_A_* − *C_B_*, *R_p_* = *P_H_*/*P_L_* as depicted in Figure 7.

## Data Availability

The original contributions presented in this study are included in the article/Supplementary Materials. Further inquiries can be directed to the corresponding author.

## Supplementary Materials

The following supporting information can be downloaded at: https://www.mdpi.com/article/10.3390/ma18245482/s1, Figure S1: Heat treatment process diagram and equipment. Figure S2: Laves phase structure of ZrFe_2_-based alloys as a function of valence electron concentration (*e*/*a*) and atomic radius ratio (*R*_A_/*R*_B_). Figure S3: XRD patterns of Zr_0.8_Ti_0.2_Fe_1.9−*x*_Ni*_x_*V_0.1_ (a) and Zr_1−*y*_Ti*_y_*Fe_1.2_Ni_0.7_V_0.1_ (b) alloys quenched under 1423 K. Figure S4: SEM image and EDS mappings of the Zr_0.8_Ti_0.2_Fe_1.9−*x*_Ni*_x_*V_0.1_ (*x* = 0.7, 0.8, 0.9) and Zr_1−*y*_Ti*_y_*Fe_1.2_Ni_0.7_V_0.1_ (*y* = 0.1, 0.3) alloys quenched under 1423 K. Figure S5: XRD patterns of Zr_0.8_Ti_0.2_Fe_1.9−*x*_Ni*_x_*V_0.1_ (a) and Zr_1−*y*_Ti*_y_*Fe_1.2_Ni_0.7_V_0.1_ (b) alloys after hydrogen absorption/desorption process. Figure S6: PCI curves of 5# alloy at 283 K (a) and 353 K (b). Table S1: Alloy systems with ZrFe_2_ Laves phase formation pattern. Table S2: Structure parameters of #5–#8 alloys before and after hydrogen absorption/desorption. References [31,33,45,46,47,48,49,50] are cited in the Supplementary Materials.
