# Supplementary material for: C15-Structured Zr-Ti-Fe-Ni-V Alloys for High-Pressure Hydrogen Compression
Source: Materials (Basel). 2025 Dec 5;18(24):5482. doi: 10.3390/ma18245482 (PMC12734445; doi:10.3390/ma18245482)
Supplement: Supplementary file 1 [file materials-18-05482-s001.zip › materials-3988869-supplementary.pdf]

Supplementary File

C15-Structured Zr-Ti-Fe-Ni-V Alloys for High-Pressure Hydrogen Compression

Jie Xu [1], Changsheng Qin [2] and Hui Wang [1,*]

1. *Guangdong Provincial Key Laboratory of Advanced Energy Storage Materials, School of Materials Science and Engineering, South China University of Technology, Guangzhou 510640, China; xu98525@163.com*
2. *Guangxi Key Laboratory of Advanced Structural Materials and Carbon Neutralization, School of Materials and Environment, Guangxi Colleges and Universities Key Laboratory of Eco-Friendly Materials and Ecological Restoration, Guangxi Minzu University, Nanning 530105, China; qcs501@126.com*

Corresponding author: Hui Wang, Email: mehwang@scut.edu.cn

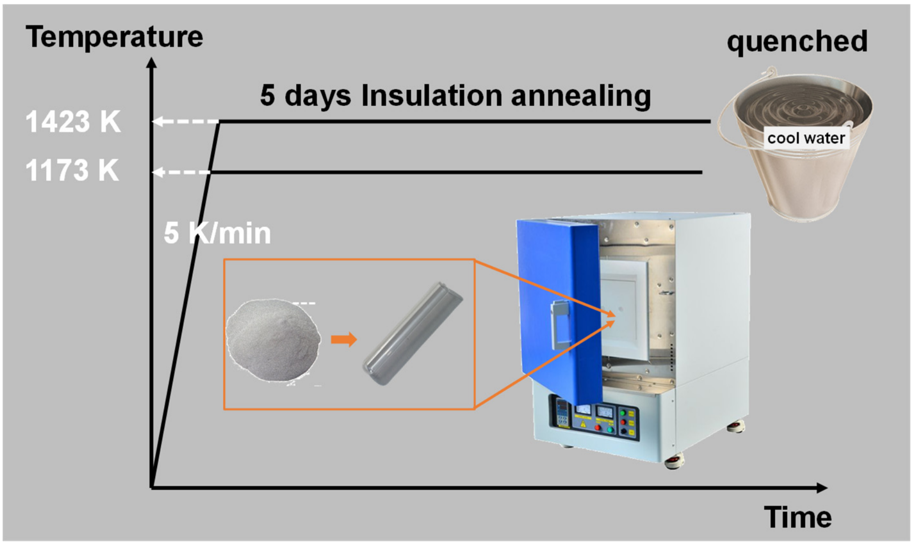

Figure S1. Heat treatment process diagram and equipment.

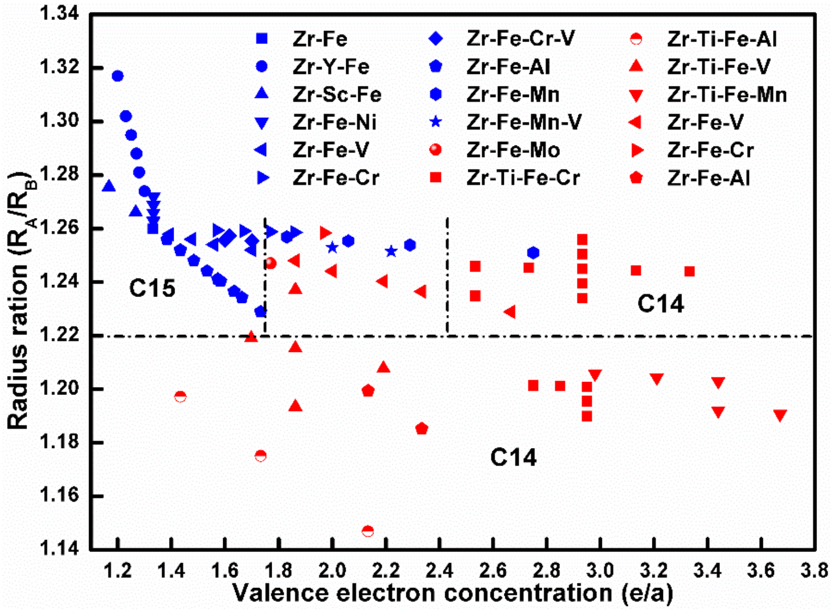

Figure S2. Laves phase structure of $ZrFe_2$-based alloys as a function of valence electron concentration (*e/a*) and atomic radius ratio (R_A/R_B).

Table S1. Alloy systems with $ZrFe_2$ Laves phase formation pattern.

Alloy	*e/a*	R_A/R_B	Phase	Ref.	Alloy	*e/a*	R_A/R_B	Phase	Ref.
$ZrFe_2$	1.33	1.260	C15	[45]	$Zr_{1.05}Fe_{1.85}Cr_{0.1}V_{0.05}$	1.62	1.257	C15	[48]
$Zr_{0.9}Y_{0.1}Fe_2$	1.3	1.274	C15	[31]	$Zr_{1.05}Fe_{1.85}Cr_{0.075}V_{0.075}$	1.61	1.257	C15	[48]
$Zr_{0.85}Y_{0.15}Fe_2$	1.28	1.281	C15	[31]	$Zr_{1.05}Fe_{1.85}Cr_{0.05}V_{0.1}$	1.60	1.256	C15	[48]
$Zr_{0.8}Y_{0.2}Fe_2$	1.27	1.288	C15	[31]	$Zr_{1.05}Fe_{1.8}Cr_{0.1}V_{0.1}$	1.70	1.255	C15	[48]
$Zr_{0.75}Y_{0.25}Fe_2$	1.25	1.295	C15	[31]	$(Zr_{0.5}Ti_{0.5})_{1.05}Fe_{1.1}Mn_{0.9}$	3.44	1.192	C14	[33]
$Zr_{0.7}Y_{0.3}Fe_2$	1.23	1.302	C15	[31]	$(Zr_{0.5}Ti_{0.5})_{1.05}FeMn$	3.67	1.191	C14	[33]
$Zr_{0.6}Y_{0.4}Fe_2$	1.2	1.317	C15	[31]	$Zr_{1.05}Fe_{1.8}Mn_{0.2}$	1.83	1.257	C15	[33]
$Zr_{0.5}Sc_{0.5}Fe_2$	1.17	1.276	C15	[45]	$Zr_{1.05}Fe_{1.7}Mn_{0.3}$	2.06	1.255	C15	[33]
$Zr_{0.8}Sc_{0.2}Fe_2$	1.27	1.266	C15	[45]	$Zr_{1.05}Fe_{1.6}Mn_{0.4}$	2.29	1.254	C15	[33]
$ZrFe_2V_{0.05}$	1.39	1.258	C15	[46]	$Zr_{1.05}Fe_{1.4}Mn_{0.6}$	2.75	1.251	C15	[33]
$ZrFe_{1.95}V_{0.1}$	1.48	1.256	C15	[46]	$Zr_{1.05}Fe_{1.7}Mn_{0.2}V_{0.1}$	2.00	1.253	C15	[33]
$ZrFe_{1.9}V_{0.15}$	1.56	1.254	C15	[46]	$Zr_{1.05}Fe_{1.6}Mn_{0.3}V_{0.1}$	2.22	1.251	C15	[33]
$Zr(Al_{0.12}Fe_{0.88})_2$	1.57	1.241	C15	[47]	$Zr_{0.5}Ti_{0.5}(Fe_{0.95}Al_{0.05})_2$	1.43	1.197	C14	[49]
$Zr(Al_{2/12}Fe_{10/12})_2$	1.66	1.234	C15	[47]	$Zr_{0.5}Ti_{0.5}(Fe_{0.8}Al_{0.2})_2$	1.73	1.175	C14	[49]
$Zr(Al_{0.2}Fe_{0.8})_2$	1.73	1.229	C15	[47]	$Zr_{0.5}Ti_{0.5}(Fe_{0.6}Al_{0.4})_2$	2.13	1.147	C14	[49]
$Zr(Al_{0.4}Fe_{0.6})_2$	2.13	1.199	C14	[47]	$(Zr_{0.9}Ti_{0.1})_{1.04}Fe_{1.7}V_{0.3}$	1.86	1.237	C14	[50]
$Zr(Al_{0.5}Fe_{0.5})_2$	2.33	1.185	C14	[47]	$(Zr_{0.7}Ti_{0.3})_{1.04}Fe_{1.7}V_{0.3}$	1.86	1.215	C14	[50]
					$(Zr_{0.5}Ti_{0.5})_{1.04}Fe_{1.7}V_{0.3}$	1.86	1.193	C14	[50]

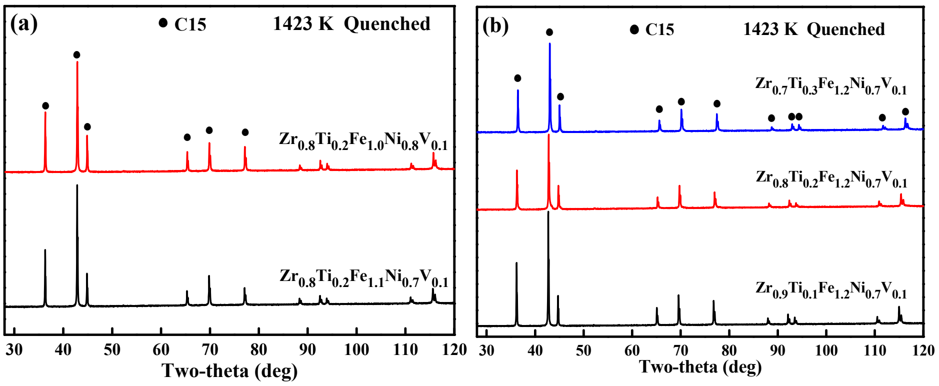

Figure S3. XRD patterns of $Zr_{0.8}Ti_{0.2}Fe_{1.9\text{-}x}Ni_xV_{0.1}$ (a) and $Zr_{1\text{-}y}Ti_yFe_{1.2}Ni_{0.7}V_{0.1}$ (b) alloys quenched under 1423 K.

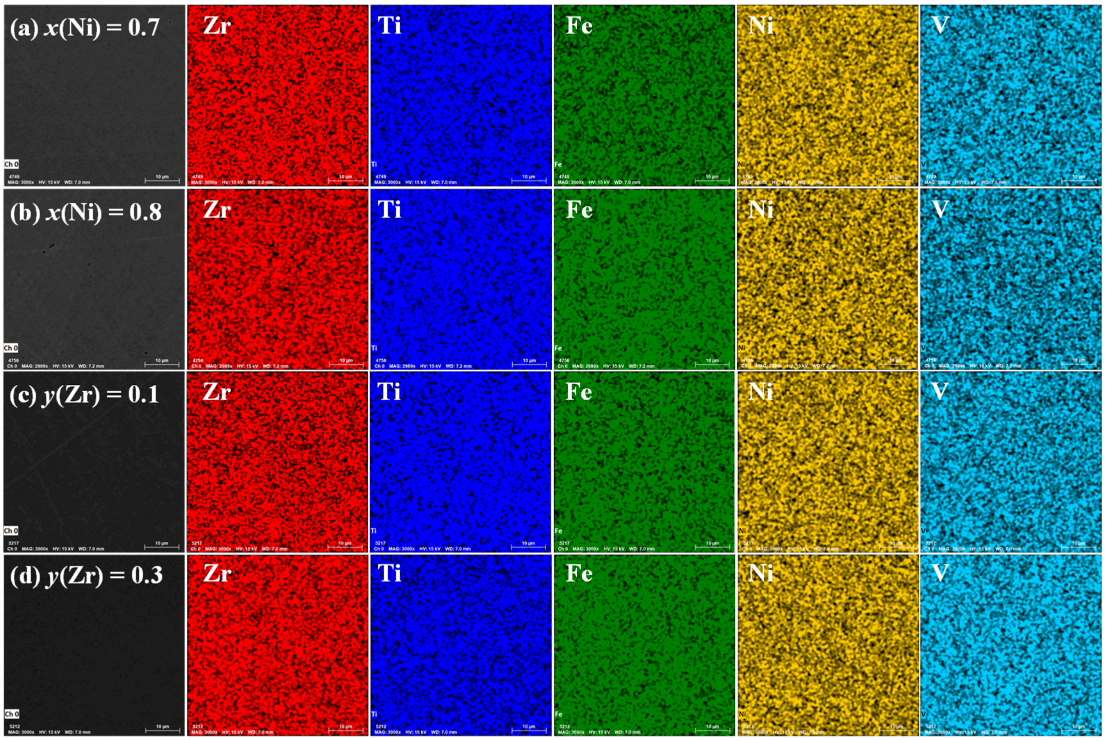

Figure S4. SEM image and EDS mappings of the $Zr_{0.8}Ti_{0.2}Fe_{1.9\text{-}x}Ni_xV_{0.1}$ (x = 0.7, 0.8, 0.9) and $Zr_{1\text{-}y}Ti_yFe_{1.2}Ni_{0.7}V_{0.1}$ (y = 0.1, 0.3) alloys quenched under 1423 K.

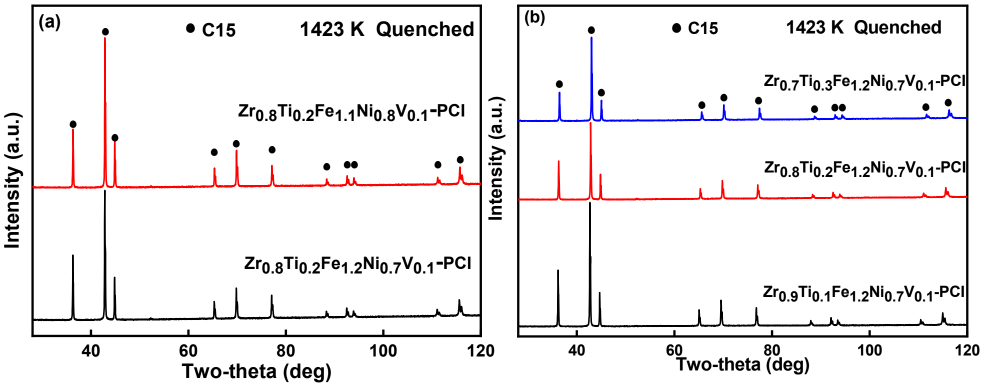

Figure S5. XRD patterns of $Zr_{0.8}Ti_{0.2}Fe_{1.9-x}Ni_xV_{0.1}$ (a) and $Zr_{1-y}Ti_yFe_{1.2}Ni_{0.7}V_{0.1}$ (b) alloys after hydrogen absorption/desorption process.

Table S2. Structure parameters of #5–#8 alloys before and after hydrogen absorption/desorption

Alloys	PCI	*Phase*	*a* (Å)	*V* (Å^3)	Δ*V*
#5	before	C15	6.9926	341.92	0.038%
	after	C15	6.9917	341.79	
#6	before	C15	6.9879	341.22	0.006%
	after	C15	6.9880	341.24	
#7	before	C15	7.0173	345.45	0.032%
	after	C15	7.0174	345.56	
#8	before	C14	6.9674	338.23	0.047%
	after	C15	6.9663	338.07	

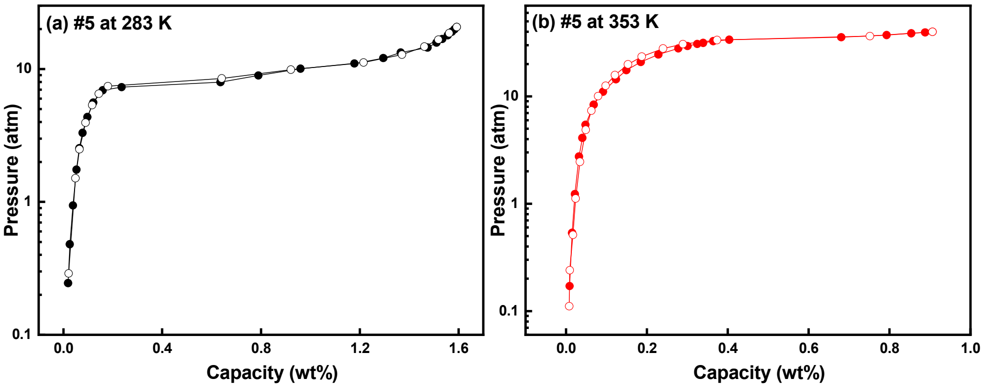

Figure S6. PCI curves of 5# alloy at 283 K (a) and 353 K (b).
